# Resistance to *Rhabdoviridae* Infection and Subversion of Antiviral Responses

**DOI:** 10.3390/v7072794

**Published:** 2015-07-07

**Authors:** Danielle Blondel, Ghizlane Maarifi, Sébastien Nisole, Mounira K. Chelbi-Alix

**Affiliations:** 1 Institute for Integrative Biology of the Cell (I2BC), Université Paris-Saclay, CEA, CNRS UMR 9198, Université Paris-Sud, Gif-sur-Yvette 91190, France; E-Mail: danielle.blondel@vms.cnrs-gif.fr; 2 INSERM UMR-S 1124, Université Paris Descartes, Centre Interdisciplinaire Chimie Biologie-Paris (FR 3567, CNRS), 75270 Paris Cedex 6, France; E-Mails: ghizlane.maarifi@parisdescartes.fr (G.M.); sebastien.nisole@inserm.fr (S.N.)

**Keywords:** interferon, rhabdoviruses, ISG, rabies virus, vesicular stomatitis virus

## Abstract

Interferon (IFN) treatment induces the expression of hundreds of IFN-stimulated genes (ISGs). However, only a selection of their products have been demonstrated to be responsible for the inhibition of rhabdovirus replication in cultured cells; and only a few have been shown to play a role in mediating the antiviral response *in vivo* using gene knockout mouse models. IFNs inhibit rhabdovirus  replication at different stages via the induction of a variety of ISGs. This review will discuss how individual ISG products confer resistance to rhabdoviruses by blocking viral entry, degrading single stranded viral RNA, inhibiting viral translation or preventing release of virions from the cell. Furthermore, this review will highlight how these viruses counteract the host IFN system.

## 1. Introduction

The establishment of an antiviral state in cells has resulted in the discovery of interferons (IFNs), and this antiviral activity is still their defining property. IFNs act on target cells to confer resistance to viral infection at various stages of viral replication; including entry, transcription, RNA stability, translation, maturation, assembly and release. The antiviral activities of IFNs are mediated by the induction of IFN-stimulated gene (ISG) products. One interesting property of ISG-mediated antiviral activity is the magnitude by which a single IFN effector can inhibit virus replication. In this review, rhabdovirus replication, IFN production, IFN signaling, ISGs conferring resistance to rhabdoviruses and the mechanisms by which these viruses counteract the IFN pathway will be described.

## 2. Rhabdoviruses

Rhabdoviruses (order *Mononegavirales*) are pathogens with a particularly broad host range among a great diversity of organisms including plants, insects, fish, mammals, reptiles and crustaceans. They are associated with significant pathogenicity in humans and livestock [1]. The prototypes of this family are vesicular stomatitis virus (VSV), a member of the *Vesiculovirus* genus, and rabies virus (RABV), a member of the *Lyssavirus* genus. VSV is an arthropod-borne virus that primarily affects rodents, cattle, swine and horses. It can induce mild symptoms upon infection of humans and other species and may also cause severe foot- and mouth-like disease in cattle and pigs. The closely related Chandipura virus has recently been associated with outbreaks of fatal acute encephalitis in several parts of India (for a review see [2]). RABV is a prototype neurotropic virus that causes fatal disease in humans and animals. Human rabies is a zoonosis, which still accounts for 50,000 deaths per year worldwide despite the availability of effective vaccines.

Although VSV and RABV have many similarities, they use different strategies to regulate their replication in the host cell, leading to the various pathologies caused by these viruses. VSV replicates rapidly, developing high levels of progenies in a minimum amount of time and strongly interferes with the host’s cell metabolism. VSV inhibits host gene expression and translation [3,4,5], interferes with the host cell’s innate immune response and induces apoptosis of infected cells [6]. In contrast, RABV replicates more slowly, has a reduced interference with the host cell metabolism and is less cytopathic than VSV [7].

Rhabdovirus virions have a bullet-like shape, conical at one end and flat at the other, with a diameter of 75 nm and a length of 100–300 nm [8,9]. The genome consists of a negative sense, single stranded RNA molecule of approximately 11 to 16 kb that encodes the five viral proteins common to all *mononegavirales* in the order of 3′-N-P-M-G-L-5′. The genome of many rhabdoviruses encodes accessory proteins, whose functions are not yet fully understood. These may occur as alternative or overlapping open reading frames (ORFs) within the major protein genes or as independent ORFs between the genes [10]. The viral RNA is associated with the nucleoprotein (N) to form a helical nucleocapsid (N-RNA). The nucleocapsid contains a significant amount of phosphoprotein (P), some of which is bound to the viral RNA-dependent RNA polymerase (RdRp), the large (L) protein. The N-RNA, together with the viral polymerase complex (P and L proteins) form the ribonucleoprotein (RNP). The RNP is enwrapped in a lipid bilayer, which is acquired from host cell membrane during the budding process. The matrix protein (M) and the glycoprotein (G) are membrane-associated proteins, whereby M is located beneath the viral membrane and maintains the compact structure of the virion by associating with both the nucleocapsid and the lipid bilayer, while the G protein is an integral transmembrane protein involved in viral entry.

**Figure 1:**
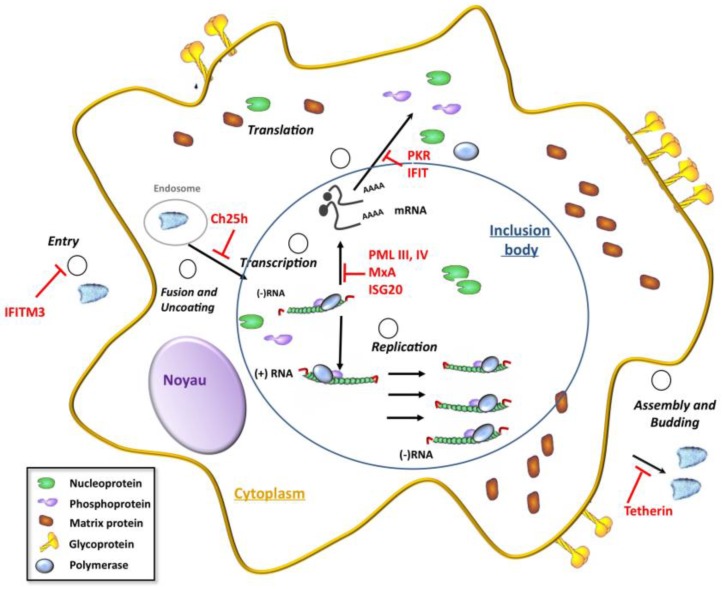
The different steps of the rhabdovirus cycle inhibited by interferon (IFN)-stimulated gene (ISG) products. IFN-inducible transmembrane (IFITM) proteins block viral entry, CH25h impairs the virus cell-fusion step by inducing cellular membrane changes, MxA inhibits primary transcription, ISG20 and ProMyelocytic Leukemia (PML) inhibit secondary transcription, protein kinase (PKR) and IFIT proteins inhibit viral translation and Tetherin prevents release of virions from the cell.

The life cycle of rhabdoviruses is entirely cytoplasmic (Figure 1). After binding to its receptor(s), the virus enters the cell via the endocytic pathway. The acidic environment within early endosomes induces a change in the conformation of the G protein that results in the fusion of the viral envelope with the endosomal membrane. After the fusion event, uncoating of the viral genome results in the release of the viral genomic RNA into the cytoplasm. The RNP constitutes the template for transcription of viral genes and the viral RNA polymerase (the L-P complex) is responsible for replication of the viral genome. RABV transcription and replication take place within Negri bodies, which are characteristic cytoplasmic inclusion bodies formed during viral infection [11]. Similar cytoplasmic inclusions have recently been reported in cells infected with VSV; these inclusions also contain the viral replication machinery [12]. During transcription, a positive-stranded leader RNA, uncapped and non-polyadenylated, and five capped and polyadenylated mRNAs encoding the five viral proteins are synthesized. At a later stage of infection, the polymerase switches to replication of the viral genome, which yields nucleocapsids containing the full-length antigenome (sense) RNA, which in turn serves as template for the synthesis of the (antisense) RNA genome. During their synthesis, both the nascent antigenome and the genome are encapsidated by the N protein. Specific transfer of the N protein to viral RNAs rather than to the cellular mRNAs is mediated by the P protein, which acts as a chaperone by binding the N alone (N°) and maintaining its solubility [13]. The neo-synthesized genomic RNPs then serve either as templates for additional rounds of transcription and/or replication, or are transported to the cell membrane where they are assembled with the M and G proteins into virions, which are then released from the cell through the budding process.

## 3. IFN Production upon Viral Infection

**Figure 2:**
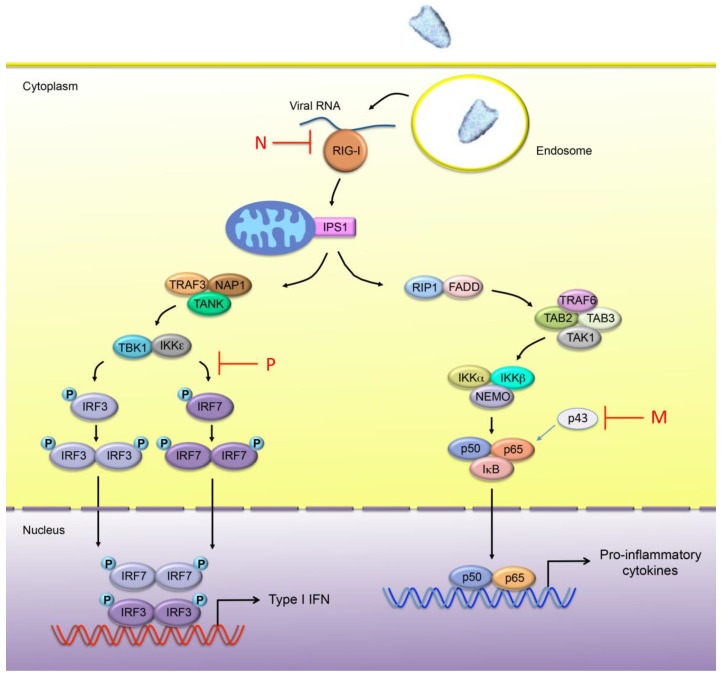
Innate immune sensing of rhabdovirus infection. In rhabdovirus-infected cells, the viral RNA is mainly detected by RIG-I. Once activated, RIG-I binds to the CARD containing adaptor protein IPS-1 (also known as MAVS, CARDIF or VISA), which phosphorylates IRF3 and/or IRF7 through TRAF3, NAP1 and TBK1/IKKε. Phosphorylated IRF3 and IRF7 homodimerize and translocate into the nucleus where they induce the expression of Type I IFN genes. IPS-1 also interacts with FADD, a death domain-containing adapter involved in death receptor signaling, and RIP1, which induces the activation of the NF-κB pathway. NF-κB is composed of homo- and heterodimeric complexes of members of the Rel family. The most common and best-characterized form of NF-κB is the p65/p50 heterodimer. A new member RelAp43 (p43) of the NF-κB family has been recently identified. Activation of the IκB kinase (IKK) complex, consisting of catalytic kinase subunits (IKKα and/or IKKβ) and the regulatory non-enzymatic scaffold protein NEMO, results in the phosphorylation and subsequence degradation of IκB. This enables free NF-κB to translocate to the nucleus, where it induces target gene expression, including pro-inflammatory cytokine encoding genes. The steps inhibited by the RABV-N and -P proteins, as well as VSV M protein are indicated in the diagram above.

Infection by rhabdoviruses triggers a cellular response through the activation of pattern recognition receptors (PRRs), leading to the production and secretion of IFN and pro-inflammatory cytokines. Two major classes of PRRs are involved in the detection of pathogens: Toll-like receptors (TLRs) and retinoic acid-inducible gene I (RIG-I)-like receptors (RLRs). These sensors can detect exogenous pathogens via the recognition of pathogen-associated molecular patterns (PAMPs). In the case of viral infection, the major PAMPs consist of nucleic acids. Although the diverse PRRs induce distinct signaling pathways, their initiation results in the activation of the nuclear factor κB (NF-κB) and IFN regulatory factor (IRF) 3 and/or IRF7. Once phosphorylated, the IRF3 and IRF7 homodimers as well as NF-κB translocate into the nucleus and induce the activity of the promoter regions of Type I IFNs and pro-inflammatory cytokines, respectively (Figure 2).

Among the TLR family, a subgroup of endosome localized TLRs (TLR3, 7, 8, 9) detect nucleic acids, whereas the other members, expressed at the cell surface, mainly recognize bacterial cell wall components or viral proteins. TLR3 detects double-stranded (ds) RNA, while TLR7 and TLR8 recognize single-stranded (ss) RNA and TLR9 recognizes unmethylated DNA with CpG motifs. Unlike membrane-bound TLRs, RLRs reside in the cytoplasm and recognize cytoplasmic RNA species. Although TLR7 and TLR9 are essential for the recognition of incoming viruses in plasmacytoid dendritic cells (pDCs), viral replication could stimulate IFN production through RLRs [14,15]. The majority of other cell types also recognize viral RNA in the cytoplasm through RLR-induced sensing. Whereas TLR7 and TLR9 are essential for the recognition of viral nucleic acids in plasmacytoid dendritic cells (pDCs), most other cell types recognize viral RNA through RLRs. The two major RLRs, RIG-I and MDA5, respond to distinct RNA virus species [16]. Indeed, whereas MDA5 is critical for picornavirus detection, RIG-I is able to sense many RNA viruses, including rhabdoviruses (Figure 2). It was demonstrated that uncapped 5′-triphosphate RNA serves as the molecular signature for the detection of viral infections by RIG-I [15,17]. Although RIG-I appears to constitute the main PRR implicated in rhabdovirus sensing, TLR3 and TLR7 may also be involved, although their exact contribution remains unclear. In the case of TLR3, it was first shown that TLR3-deficient mice were as capable as wild-type to clear VSV infection [18]. However, TLR3−/− mice were found to display a reduced susceptibility to a pathogenic RABV strain [19], which could be attributed to the involvement of TLR3 in the formation of viral Negri bodies rather than to its implication in IFN response [19]. TLR7 was found to be involved in VSV sensing in mice [20], whereas it appeared not to be associated with RABV infection; neither in mice [21] nor in human pDCs [15].

Virus-induced IFN production is tightly regulated and the aberrant production of this cytokine is harmful or even fatal to the host. Post-translational modifications of the transcription factors IRF3 and IRF7 are central regulatory mechanisms of Type I IFN mediated antiviral response. It has been reported that VSV infection triggers small ubiquitin modifier (SUMO)-ylation of IRF3 and IRF7 leading to the negative regulation of Type I IFN gene expression [22]. Accordingly, SUMOylation deficient IRF3 and IRF7 mutants lead to higher levels of IFN mRNA induction after viral infection. In contrast, increased MDA5 or RIG-I SUMOylation is correlated with elevated Type I IFN induction [23,24].

## 4. IFN Signaling and ISGs Conferring Resistance to Rhabdoviruses

### 4.1. IFN Signaling

The establishment of an antiviral state in cells is the defining function of IFNs and the property that resulted in their discovery in 1957 by Isaacs and Lindenmann [25]. Any stage in viral replication may be a target for inhibition by IFNs; including entry, transcription, RNA stability, translation, maturation, assembly and release. Three classes of IFNs have been identified and are designated Types I to III. In humans, Type I IFNs include IFNα, IFNβ, IFNε, IFNκ and IFNω. A single gene encodes each Type I IFN, with the exception of IFNα, which is comprised of 13 subtypes. IFNγ is the sole Type II IFN, and Type III IFNs are composed of IFNλ1, IFNλ2, IFNλ3 and IFNλ4 [26,27].

The interaction of Type I IFN with IFNAR leads to the activation of the JAK tyrosine kinases (Tyk2 and JAK1) that in turn phosphorylate STAT1 and STAT2. Phosphorylated STAT1 and STAT2 heterodimerize and form, with the DNA binding protein IFN regulatory factor 9 (IRF9), a complex called IFN-stimulated growth factor 3 (ISGF3). There is also growing evidence that Type I IFN activates a STAT2/IRF9 complex that forms an ISGF3-like in the absence of STAT1 [28,29]. ISGF3 translocates to the nucleus and binds to ISG promoters harboring an IFN-stimulated response element (ISRE). The binding of Type II IFN to its receptor, IFNGR, results in the phosphorylation of STAT1 by JAK1 and JAK2. STAT1 homodimers migrate to the nucleus and bind to a DNA element termed GAS (gamma-activated sequence) in the promoter regions of ISGs. Finally, Type III IFNs that are structurally and genetically distinct from Type I IFNs, bind to a different receptor, IFNLR1, but activate the same signal transduction pathway [26]. Expression of IFNLR1 is largely restricted to epithelial cells [30]. Consequently, a large number of cell types respond very poorly or have no response at all to Type III IFNs.

The transcriptional regulation of effector genes downstream of the Jak/STAT pathway contributes to pleiotropic responses induced by IFNs [26]. The products of some ISGs have been shown to mediate the antiviral effects of IFNs. Interestingly, microRNAs (miRNA) induced by IFNs emerged as novel regulators of viral infection [31]. Additionally, miRNAs negatively regulate the RIG-I antiviral pathway. Furthermore, VSV infection increases miR-146a expression within mouse macrophages in a RIG-I-NF-κB-dependent manner. In turn, miR-146a negatively regulates VSV-triggered Type I IFN production, thus promoting VSV replication in macrophages [32]. This aspect will not be discussed in more detail in the review.

### 4.2. ISG Products Conferring Resistance to Rhabdoviruses

The antiviral restriction factors, which are cellular proteins that confer viral resistance, are themselves encoded by ISGs. Therefore, the ISG products exhibiting antiviral activity are also known as restriction factors and can be implicated in both intrinsic and innate immune activities. They act cooperatively to confer viral resistance by inhibiting various stages of viral replication. Some of these factors are highly constitutively expressed and are able to exert an intrinsic antiviral activity independently of IFN, whereas their innate immune activity requires a pathogen recognition event and/or induction by IFN.

Until 1999, three IFN-responsive antiviral proteins had been considered to be involved in the antiviral processes: the Mx (for myxovirus resistance) dynamins, the double-stranded RNA-dependent protein kinase (PKR) and the 2′-5′ oligoadenylate synthetases that function through RNase L. The demonstration that IFN confers resistance to viral infection in cells derived from mice triply deficient (TD) in PKR, Mx1 and RNase L [33] revealed the implication of other antiviral pathways.

IFN treatment induces the expression of hundreds of ISGs, but only a selection of their products have been demonstrated to be responsible for the inhibition of VSV replication. Indeed, ISG products such as PKR [34], Mx [35], p53 [36], ISG20 [37], *Ifit2*/ISG54 [38], IFITM3 [39], Tetherin [39], Ch25h [40] and ProMyelocytic Leukemia (PML) [41,42,43] have been reported to confer resistance to VSV infection (Figure 1). Although PKR and the 2′-5′ oligoadenylate synthetases have been shown to be implicated in antiviral defense, their overexpression has not been reported to alter VSV infection.

Recent ectopic expression and gene silencing experiments have identified many novel ISGs with inhibitory activity against viruses from various families [26,44]. In the case of VSV, 34 ISG products have been shown to elicit an antiviral effect [45], which include Mx, Ch25h, IRF1, IFITM3, ISG20 and some members of TRIM protein family (PML/TRIM19, TRIM25, TRIM31, TRIM62).

IFNs inhibit VSV replication at different stages. Individually, some ISG products can interfere with a particular stage of the VSV life cycle. For example IFITM proteins block viral entry [39], CH25h impairs the virus cell-fusion step by inducing cellular membrane changes [40], MxA inhibits primary transcription [35,46], ISG20, a 3′-5′ exonuclease, degrades single stranded viral RNA [37], PML inhibits secondary transcription [41,42], PKR [47] and IFIT [38] proteins inhibit viral translation and Tetherin prevents release of virions from the cell [39] (Figure 1). PML and IFITM3 have been reported to confer resistance to RABV [48,49,50], however the effect of the other human antiviral mediators on RABV is still unknown. The antiviral ISG mediators, also known as host restriction factors, reside in the cytoplasm, the nucleus, the plasma membrane and the viral particle itself. Their localization within the cell often corresponds to the stage of the virus life cycle with which it interferes. In addition to having an intrinsic anti-VSV activity, various ISG products, such as some TRIM proteins have been recognized as important immune signaling mediators [42,51].

One interesting property of ISG-mediated antiviral activity is the magnitude with which a single IFN effector can inhibit virus replication (Table 1). Usually ISGs are known to exert antiviral functions. Intriguingly the IFN-inducible gene IFI35 (also known as IFP35) was identified as factor required for VSV infection [52]. IFI35 negatively regulates RIG-I activation and also mediates its proteasomal degradation through ubiquitination [53]. Whether the requirement of IFI35 is specific to VSV infection is still unknown. This will not be further developed in this review.

#### 4.2.1. IFITM

The IFN-inducible transmembrane (IFITM) genes are enhanced by cell treatment with IFNs since they contain the ISREs in their promoter. IFITM proteins belong to the CD225 protein superfamily, which can be found in nearly every domain of life, ranging from bacteria to invertebrates to primates. In humans, there are at least four functional members of IFITM proteins: IFITM1, IFITM2 and IFITM3, previously named 9-27, 1-8D and 1-8U, respectively, are expressed in a variety of tissues; IFITM5 is limited to the bone. IFIM4P is a pseudogene (reviewed in [54]). Mouse *IFITM1*, *IFITM2*, *IFITM3* and *IFITM5* genes are orthologues to their human counterparts. In addition, mice have two other *IFITM* genes: *IFITM6* and *IFITM7*.

IFITM proteins are a group of small ISGs (15 kDa) that have been reported to inhibit infection of Influenza A virus, West Nile virus and Dengue virus [55,56]. These proteins inhibit viral membrane fusion, thus resulting in cellular protection from a diverse range of infections. An early observation of IFITM proteins controlling viral infection was reported in 1996 [57], where overexpression of IFITM1 was shown to inhibit VSV replication, although less potently than the IFN-induced protein MxA [57]. This study is the first description of the antiviral activity of an IFITM protein.

All IFITM proteins have been shown to restrict cell entry of many viruses [54,56,58,59,60]. However, IFITM3 is the most potent IFITM family member in restricting viral replication in cell culture [61]. IFITM3 inhibits a VSV entry step following endocytosis, but at or before the point of primary transcription of incoming viral genomes [39] (Table 1). Both the N-terminal 21 amino acid residues and C-terminal transmembrane region of IFITM3 are required for its antiviral function. In addition, IFITM3 has been shown to inhibit an early stage of the RABV replication cycle by targeting entry mediated by the viral glycoprotein [50].

#### 4.2.2. Ch25h

The cholesterol-25-hydroxylase (Ch25h) has been identified as a broadly antiviral ISG through a systematic functional screen [40]. It has been shown that Ch25h inhibits growth of VSV and a wide-range of enveloped viruses through the production of a soluble antiviral factor that is not IFN. Ch25h is an endoplasmic-reticulum-associated enzyme that catalyzes the oxidation of cholesterol into the soluble component 25 hydroxycholesterol (25HC) [62]. The viral restriction mediated by 25HC occurs at an early stage, resulting in a 1 log inhibition of viral production (Table 1) [40]. The overexpression of factors controlling sterol biosynthesis or the addition of intermediates in the sterol biosynthesis pathway, such as mevalonate, do not rescue 25HC-mediated virus inhibition; suggesting that 25HC blocks membrane fusion. Liu *et al.* [40] proposed that 25HC impaired viral entry at the virus cell-fusion step by inducing cellular membrane changes due to membrane expansion or aggregation [40]. This inhibition is not specific to a particular structural class of viral fusion proteins (such as Class I, II or III) and is not linked to either pH-dependent or pH-independent fusion processes, but is due to membrane perturbation resulting from the oxidation of the cholesterol. However, the authors do not exclude that the Ch25h protein may have additional antiviral mechanisms to 25HC, perhaps through its association with the endoplasmic reticulum.

#### 4.2.3. MxA

Humans harbor two *M*x genes (*MX1* and *MX2*) in the chromosome 21 [63], which are induced only in response to Type I or Type III IFNs via the ISRE present in their gene promoter [64]. Human *Mx1* and *Mx2* gene products are MxA and MxB, respectively. Mx proteins are classified as large dynamin-like GTPases due to their similarities to dynamins [65]. MxA and dynamins share structural and functional aspects including domain organization, GTPase activity and homo-oligomerization capacity [66]. The Mx proteins inhibit several different viruses by blocking early stages of their replication cycle [65,67].

The human MxA and MxB proteins [35], like those of the rat (Mx2 or Mx3) [68] and the mouse (Mx2) [69], are cytoplasmic, while the Mx1 protein has a speckled nuclear localization in both mouse and rat cells. Mx proteins from different species exhibit distinct antiviral activities with a specificity conferred by their subcellular localization [65,67]. In general, nuclear Mx proteins inhibit viruses that replicate in the nucleus such as influenza virus, whereas cytoplasmic forms inhibit VSV and other RNA viruses that replicate in the cytoplasm.

Rat Mx1, mouse Mx2 and human MxA are active against VSV. More recently, it has been shown that bovine Mx1 inhibits replication of the rabies virus [70]. VSV is among the viruses that are strongly repressed by human MxA. Cells overexpressing MxA acquire a high degree of resistance to VSV in a 3 logs reduction in viral titers compared to wild-type cells [35] (Table 1). MxA inhibits VSV primary transcription, suggesting that MxA alters VSV polymerase function [46]. An interaction between MxA and VSV proteins has not yet been demonstrated.

#### 4.2.4. PML/TRIM19

PML (also named TRIM19 for TRIpartite Motif protein 19) belongs to the TRIM family that comprises over 70 members whose functions span a broad array of physiological processes, including cell proliferation, differentiation and antiviral defense [71]. PML is the organizer of small nuclear-matrix structures named nuclear bodies (NBs) [72]. In response to diverse stimuli, PML NBs recruit a growing number of proteins implicated in different cellular processes including apoptosis, senescence, protein degradation and antiviral defense [73,74,75,76]. PML is covalently conjugated to small ubiquitin modifier (SUMO) [77]. This modification, which is required for PML NB functions, alters PML localization, stability and capacity to interact with other partners.

Several PML isoforms generated by alternative splicing from a single gene are designated PMLI to PMLVIIb [74,78]. They share the same N-terminal region, which encodes the RBCC/TRIM (RING finger, B-box, and Coiled-Coil) motif, but differ in their C-terminal region due to alternative splicing. The variability of the C-terminal region is important for the specific function of each PML isoform [74]. PML confers resistance to RNA and DNA viruses from different families. This has been shown in cells stably expressing individual PML isoforms or in cells depleted for PML by RNA interference (reviewed in [75,76]). The antiviral effect of PML has been observed *in vivo*, as PML deficiency renders mice more susceptible to VSV infection [43]. A study performed with all PML isoforms revealed that only PMLIII and PMLIV could confer resistance to VSV infection [42]. The anti-VSV activity of PMLIV is higher than that of PMLIII, since the overexpression of PMLIII and PMLIV results in 2 and 3 logs reduction in VSV titers, respectively [41,42] (Table 1). The anti-VSV activity of PMLIII is strictly IFN-independent, whereas resistance to VSV exerted by PMLIV occurs via two independent mechanisms. PMLIV is able to inhibit VSV replication in an IFN-independent manner and is also able to positively regulate IFNβ synthesis *via* the enhancement of IRF3 phosphorylation. Both activities of PMLIV require its SUMOylation. Among all PML isoforms, only PMLIV is able to recruit the peptidyl-prolyl isomerase (Pin1) [42], known to interact with phosphorylated IRF3 and to induce its proteasomal degradation [79]. The interaction of Pin1 with SUMOylated PMLIV leads to the recruitment of Pin1 within PML NBs and results in an increased stability of phospho-IRF3 and in an enhanced IFNβ production [42]. Further investigations are required to demonstrate how PMLIII and PMLIV exert their intrinsic anti-VSV activity by interacting with a viral or a cellular protein required for VSV replication.

In the case of RABV, among all the PML isoforms tested only PMLIV confers intrinsic resistance to this virus by inhibiting viral transcription, resulting in a 2 logs reduction of viral titer [48]. This anti-RABV effect exerted by PMLIV is independent of IFN production, as PMLIV does not increase IFN production within infected cells ([48] and unpublished data). Further investigations are needed to elucidate the mechanism of action of PMLIV against RABV.

#### 4.2.5. ISG20

IFN-stimulated gene 20 kDa protein is encoded by the human ISG20 gene. ISG20 is a 3′-to-5′ exonuclease specific for single-stranded RNA involved in host defense against RNA viruses. ISG20 is transcriptionally induced by both Types I and II IFNs [80,81]. Its induction by IFN is strictly dependent upon the activation and the binding of IRF1 to a specific ISRE on the ISG20 promoter. ISG20 is also directly induced by synthetic dsRNA via NF-κB and IRF1 activation [82].

ISG20 overexpression in HeLa cells reduces VSV mRNA and protein synthesis resulting in a 0.5 log inhibition of viral production (Table 1). This anti-VSV activity requires the exonuclease activity of ISG20. In addition, the anti-VSV activity of IFN is reduced in cells expressing the inactive exonuclease mutant form of the ISG20 protein, suggesting that the antiviral activity of IFN against VSV is partly mediated by ISG20.

#### 4.2.6. IFIT

IFIT (IFN-induced proteins with tetratricopeptide repeats) genes encode a family of cytoplasmic proteins that are induced after Type I IFN or IRF3-dependent signaling. They contain the ISREs in their promoter that are recognized by members of the IRF family of transcription factors [83]. IFN and many inducers that activate IRFs induce their expression. The strongest *IFIT* inducers are Types I and III IFNs, whereas induction by Type II IFN is much weaker. *IFIT* genes are also induced in cells infected with various RNA viruses, including VSV. All IFIT proteins contain several full and partial tetratricopeptide repeat (TPR) motifs [84], which are critical for viral regulation. By convention, the human *IFIT* genes are spelt using capital letters whereas the mouse *ifit* genes are spelt in lowercase. In humans, four members have been characterized, *IFIT1* (also named *ISG56*), *IFIT2* (also named *ISG54*), *IFIT3* (also named *ISG60*) and *IFIT5* (also named *ISG58*), whereas three members are expressed in mice, *ifit1* (also named *ISG56*), *ifit2* (also named *ISG54*) and *ifit3* (also named *ISG49*). The cognate members of two species have distinct sequences; for example, human IFIT2 and mouse Ifit2 are only 62% homologous. Thus, they share names but not equating properties.

IFIT proteins contribute to an antiviral state against some viruses through multiple mechanisms by binding components of the eIF3 translation initiation complex and inhibiting protein translation, binding viral RNA and sequestering viral RNA or proteins in the cytoplasm [85,86]. Both IFIT1 and Ifit1 can specifically bind mRNA 5′ ends whose caps lack the 2′-O-methylation of the first ribose, a specificity of some viral but not cellular mRNAs thus preventing viral mRNA translation [87]. Remarkably, IFIT1 inhibits viral translation by two different mechanisms (eIF3 and cap-RNA binding), it uses its N-terminal and central region to bind to RNA, whereas its C-terminal binds eIF3 [88,89].

Depletion of IFIT1, IFIT2 or IFIT3 in HeLa cells with siRNA results in an increase in the rate of infection by VSV. It has been reported that overexpression of IFIT3 in Vero cells results in a 1 log decrease of virus titer (Table 1) and IFIT3 depletion in human cells reduces the anti-VSV activity of IFNα [90].

#### 4.2.7. Tetherin

Tetherin is an IFN-inducible, 28- to 36-kDa transmembrane protein also known as BST2 (Bone marrow stromal Ag 2, also named CD317 or HM1.24). Tetherin is constitutively expressed in several cell types and its expression is strongly induced by all three types of IFNs [91]. Tetherin is a Type II transmembrane protein that contains an N-terminal cytoplasmic domain, a single membrane-spanning α-helix, an extracellular domain, and a C-terminal glycosylphosphatidylinositol (GPI) anchor attaching to the cell membrane lipid bilayer [92]. Tetherin associates with cholesterol-enriched lipid rafts, which are involved in both virus budding and cell-to-cell spread [93].

Expression of Tetherin in HEK293 cells confers resistance to VSV resulting in a 3 logs reduction of viral production (Table 1). Tetherin impairs a late step in the VSV replication cycle, most likely virion particle release from infected cells [39]. Tetherin depletion reduces the capacity of IFNα to inhibit VSV at lower concentrations (1 to 10 IU/mL), but the attenuation effect decreases in cells treated with high IFN concentration, probably due to the participation of other ISG products.

#### 4.2.8. GBP1

There are at least two forms of IFN-induced Guanylate Binding Proteins (GBPs) in human and murine cells [94]. They have an affinity for guanylate and function as GTPases. They belong to the dynamin superfamily of large GTPases characterized by an oligomerization-dependent GTPase activity [95]. GBP1 is highly induced by IFNγ [96]. Overexpression of human GBP-1 inhibits 0.5 log VSV production, protecting cells from the subsequent cytopathic effect (Table 1). In addition, decreasing GBP-1 levels using antisense RNA to GBP1 reduces the anti-VSV activity of IFN. Further investigations are required to determine the exact viral stage that is inhibited by GBP-1 [97].

**Table 1:** Main human ISG products conferring intrinsic resistance to vesicular stomatitis virus (VSV) and rabies virus.

ISG Products	Degree of Inhibition of Viral Production	Mechanisms	References
**IFITM3**	1 log	Inhibits a VSV entry step after endocytosis.	[39]
**Ch25h**	1 log	Inhibits VSV entry by production of 25-Hydroxycholesterol.	[40]
**MxA**	3 logs	MxA confers resistance to VSV. Inhibits VSV primary transcription.	[35] [46]
**PMLIII**	2 logs	Inhibits VSV at transcriptional level.	[41]
**PMLIV**	3 logs	Inhibits VSV and rabies virus at transcriptional level.	[42]
**PMLIV**		Positively regulates IFNβ synthesis during VSV infection.	[42]
**ISG20**	0.5 log	Reduces VSV mRNA synthesis and requires its exonuclease activity.	[37]
**Tetherin**	3 logs	Inhibits viral particles release from infected cells.	[39]
**IFIT3**	1 log	Reduces VSV production.	[90]
**GBP1**	0.5 log	Reduces VSV production.	[97]

### 4.3. Results Obtained from VSV-Infected Knockout Mice and Their Derived Cell Lines

Studies using mice defective in ISGs revealed their implication in antiviral defense (Table 2). Compared to the parental mice, PKR−/− mice are more susceptible to VSV infection and die from acute infection of the respiratory tract [47]. In addition, IFNα/β are unable to rescue PKR−/− mice from VSV infection [34]. However, the overexpression of human PKR in culture cells does not alter VSV replication [98]. The generation of triply deficient (TD) PKR, Mx1 and RNase L mice showed that IFN still inhibited VSV replication in their derived cell lines revealing the existence of other pathways [33].

**Table 2:** Results obtained from VSV-infected knockout mice and their derived cell lines.

ISG	Mechanisms	References
**PKR−/− mice**	Are more susceptible to VSV infection and die from acute infection of the respiratory tract.	[47]
	IFNα/β is unable to rescue PKR−/− mice from VSV infection.	[34]
**PKR−/−, RNaseL−/−, Mx1−/− MEFs**	IFN still inhibits VSV replication revealing the existence of other pathways.	[33]
**PML−/− mice**	Have an increased susceptibility to VSV compared to parental mice	[43]
**p53−/− mice**	Are more sensitive to VSV infection compared to parental. mice.	[36]
**Ifit2 (ISG54)−/− mice**	Are very vulnerable to neuropathogenesis caused by VSV infection.	[38]
**Tetherin−/− mice**	Intranasal infection of VSV results in lower viral titers in lungs.	[99]

Ablation of the *PML* gene by homologous recombination has shown that mice are viable and are able to develop normally. However, the loss of PML alters viral replication. Analysis of PML−/− mice when compared to parental mice revealed that they were more susceptible to VSV infections [43]. Additionally, fibroblasts derived from *PML* knockout mice, PML−/− MEFs, exhibited enhanced VSV and RABV multiplication [42,48].

P53 was discovered in 1980, and its connection to the IFN pathway was revealed over 20 years later by Taniguchi and colleagues who showed that Type I IFN directly induced p53 expression via an active ISRE motif in its promoter [36]. They also reported in the same study that p53−/− mice were more susceptible to VSV compared to parental mice and that the VSV yield was more than 30-fold higher in p53−/− MEFs than in wild-type.

Using *Ifit2* (*ISG54*) knockout mice, Fensterl *et al.* showed that they were more vulnerable to neuropathogenesis caused by intranasal VSV infection compared to parental or *Ifit1*−/− mice [38]. Interestingly, resistance to VSV conferred by Ifit2 was restricted to neurons.

Tetherin−/− mice were generated and challenged with VSV [99]. Both Tetherin−/− and parental mice have similar tissue viral burdens after systemic infection with VSV. Intriguingly, only at short times post-infection did local intranasal infection of VSV result in lower viral titers in the lungs of Tetherin−/− mice compared to parental mice. Determination of IFN titers in the lung tissue of VSV-infected mice revealed that Tetherin−/− mice also had lower levels of IFNα compared with their parental counterparts [99]. This suggests that Tetherin may play a role in IFNα production.

## 5. Subversion of Antiviral Response or Viral Evasion of Host Immunity

Viruses use the cellular machinery to replicate, and they have developed various strategies to inhibit the actions of IFNs by altering IFN production, IFN signaling or antiviral mediators (reviewed in [100,101]).

Although VSV and RABV share a similar organization of their genomes, they differ in their ability to interplay with the cellular host defense system. VSV is a fast and highly cytopathic virus, which can shut down host cell gene expression to evade the host cellular antiviral response and the Matrix (M) protein plays an essential role in this process (see Section 5.1). In contrast, RABV replicates slowly and requires the host cell to remain intact; it must therefore counteract the innate antiviral response mediated by IFNs. The RABV phosphoprotein, cofactor of the RNA polymerase, is known to antagonize several stages of the IFN pathway (see Section 5.2, Section 5.3 and Section 5.4) and to disrupt PML NBs (see Section 5.5).

### 5.1. Shutdown of Host Gene Expression by VSV M

The matrix protein (M) (27 kDa) is a major structural component of the VSV particle and plays a major role in viral assembly and budding. The VSV M protein is also responsible for the cytopathic effects associated with VSV infection. The ability of VSV to counteract the host IFN system has been related to the highly cytopathogenic nature of the virus. Indeed, the differential capacity of VSV to inhibit IFN induction in distinct cell types following infection is correlated to the difference in the efficiency with which the virus is able to affect global shutoff of the host cell [102]. The VSV M protein is responsible for the cytopathic effects associated with VSV infection. Indeed, expression of M alone results in the rounding of cells [103,104], and it also inhibits cellular transcription [105]. This is done by inactivating the basal transcription factor TFIID, subsequently inhibiting Polymerase II-mediated transcription of host genes [106], however the precise molecular mechanisms underlying this inhibition are not known. Importantly, mutation of critical residues M51, E213, V221 and S226 in VSV M have been shown to abolish the ability of M to inhibit host RNA synthesis and thereby IFN production [3]. Importantly, subcellular trafficking of M appears to play roles in the disregulation of cellular functions, as the portion of M protein that is located in the nucleus blocks nuclear export of all cellular mRNAs by interacting with the nucleoporin Nup98 and the export factor Rae [107,108,109,110]. Significantly, both Nup98 and Rae are ISGs, implying that their role in nuclear export may contribute to the IFN response. Thus it has been proposed that inhibition of nuclear export by M may affect aspects of the cellular IFN response [110].

### 5.2. Inhibition of IFN Production by RABV P and N

The RABV phosphoprotein P (40 kDa) is a multifunctional protein that plays a central role in the network of viral and host protein interactions (Figure 4). First, P protein is an essential noncatalytic cofactor of the viral dependent RNA polymerase L and a regulatory protein that plays a role in viral transcription and replication. The second most important role of the RABV phosphoprotein is its ability to inhibit IFNβ induction, as demonstrated by experiments in which recombinant RABV defective for P expression lost the ability to prevent IFNβ production [111]. Specifically, P is able to block the critical phosphorylation of IRF3 by the cellular kinase TBK1 (Figure 2). Although the mechanism of this inhibition remains unclear, this inhibition results in a blockage of IFNβ production in RABV-infected cells.

It has also been shown that RABV P can inhibit phosphorylation of IRF7 by the kinase IKKi, thereby blocking the activation of ”later” IFNα subtypes which are important for propagation of the IFN response [111]. A small region of the P protein, within residues 176–186, has been shown to be important for the IRF blocking activity of RABV P. This region is also involved in viral pathogenicity *in vivo*, although the precise molecular interactions involved remain to be resolved [112].

The RABV N protein (50 kDa) encapsidates the genomic RNA [113] and thus is essential for the replication of the genome. It plays also an important role in blocking the early stages of IFN induction in infected cells. This has been shown using a recombinant chimeric rabies virus strain, CE(NiN), in which the N gene of the virulent Nishigahara strain has been inserted into the genetic background of a non-lethal attenuated derivative strain of Nishigahara (Ni-CE strain), in the place of the endogenous Ni-CE N gene. Masatani *et al.* have shown that CE(NiN) induced the expression of the IFNβ gene less efficiently than in the parental Ni-CE strain in human neuroblastoma cells; indicating that the N protein is able to inhibit the innate immune response [114]. This suggests that such a deficiency in the N protein contributes to the defective pathogenicity of the Ni-CE strain [114]. Importantly, the N protein does not act as an independent antagonist of IFN signaling and is only active in the context of viral infection. In addition, the activation of RIG-I was severely inhibited in cells infected with the Nishigahara and CE(NiN) strains, but not with the Ni-CE strain [114]. Thus, it was hypothesized that rather than acting as an inhibitor of the IFN signaling pathway, N protein acts by encapsidating the viral genomic RNA and inhibiting its recognition by RIG-I (Figure 2). Similar observations were made using other viral IFN antagonist proteins including Ebola virus VP35, which acts by masking viral PAMPs from cellular receptors [115].

### 5.3. Inhibition of the NF-κB Pathway by RABV M

NF-κB is activated by viral infections to induce the expression of antiviral cytokines. RelA, one of the members of the NF-κB family, has been proposed to be crucial for early IFNβ expression [116]. A new member RelAp43 has been recently identified [117]. Luco *et al.* [117] have shown that although RelAp43 lacks the transactivation domain, it is able to potentiate RelA-mediated transactivation and to stabilize dimers comprising p50, leading to the induction of the expression of IFNβ (Figure 2). Interestingly, RelAp43 is specifically targeted by the matrix protein of lyssaviruses resulting in the inhibition of the NF-κB pathway (Figure 2). It should be mentioned that the M protein of laboratory adapted or vaccine strains (SADB19) has lost the ability to interact with RelAp43 most likely during the virus adaptation to cell culture or during selection for vaccine preparation [117]. This suggests that the inhibition of the NF-κB pathway contributes to the pathogenesis of the virus and its escape from innate immune response.

### 5.4. Inhibition of IFN Signaling by RABV-P

The RABV-P is not the only protein derived from the P gene acting as an IFN antagonist. The P gene encodes the full length P protein, as well as four N-terminally truncated protein isoforms (P2–P5) (Figure 4), which are produced from the P mRNA transcript by a leaky ribosomal scanning mechanism [118]. Whereas P translation is initiated from the initial Methionine (Met), the shorter products are translated from the four subsequent internal in-frame Met codons [118]. In addition, the P-protein isoforms have distinct trafficking properties depending on multiple targeting signals (two Nuclear Export signal (NES), two nuclear localization signal (NLS). P1 and P2 localize mainly in the cytoplasm, while P3 accumulates in the nucleus (Figure 4) [119,120,121].

**Figure 3:**
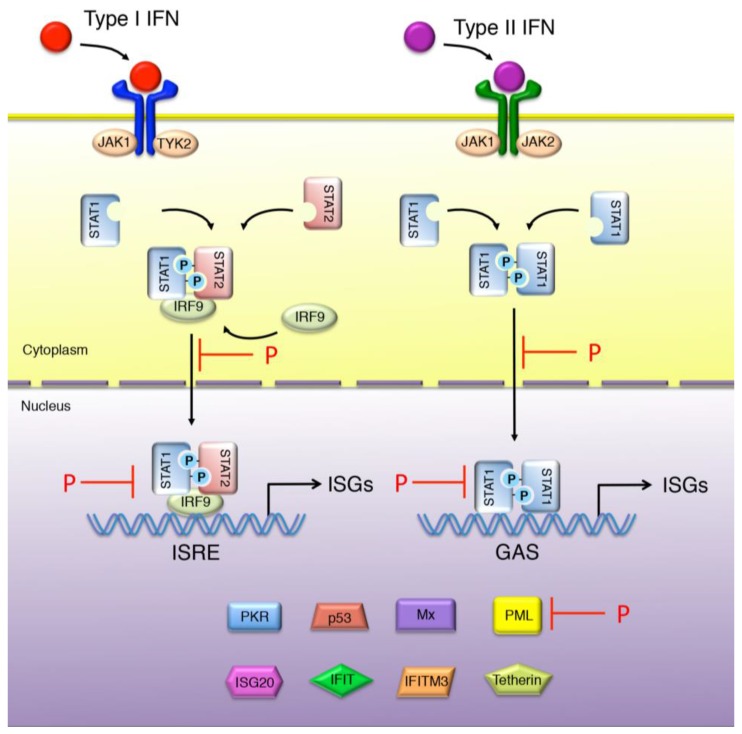
Inhibition of IFN signaling by RABV-P. The interaction of Type I IFN with IFNAR leads to the activation of the JAK tyrosine kinases (Tyk2 and JAK1) resulting in the phosphorylation of STAT1 and STAT2, which form, with IRF9, the complex ISGF3. ISGF3 translocates to the nucleus and induces the expression of ISGs that harbor an ISRE. The binding of Type II IFN to its receptor, IFNGR, results in the phosphorylation of STAT1 by JAK1 and JAK2. pSTAT1 homodimers migrate to the nucleus and bind to the GAS in the promoter region of specific ISGs. The steps counteracted by the RABV-P are indicated: P interacts with STAT1 and STAT2, and thereby blocks IFN signaling by STAT1 sequestration in the cytoplasm and the inhibition of pSTAT1 and ISGF3 binding to DNA promoters.

In addition to antagonizing IFN production (Figure 2), RABV also has a mechanism to inhibit IFN signaling (Figure 3). Therefore, virus infected cells are resistant to IFN action. RABV P interacts with STAT1 and STAT2, and thereby blocks IFN signaling through several mechanisms, including sequestration of STAT in the cytoplasm and inhibition of pSTAT1 and ISGF3 binding to DNA promoters [122,123,124]. However, RABV P neither induces STAT1 degradation nor interferes with STAT1 phosphorylation. It has been shown that the last 30 residues of P are required to bind STAT. As these residues are maintained in all five RABV P isoforms, P1-5 retain the capacity to interact with STAT1 [123]. The retention of STAT proteins in the cytoplasm is mediated by P1 and P2 proteins, which are excluded from the nucleus due to the NES signal. Indeed P1 alone can cause the cytoplasmic retention of STAT1, and treatment of cells with leptomycin B reverses or inhibits cytoplasmic sequestration of STAT1 [122]. However`, RABV P neither induces STAT1 degradation nor interferes with STAT1 phosphorylation. It has been shown that the last 30 residues of P are required to bind STAT. As these residues are maintained in all five RABV P isoforms’, P1-5 retain the capacity to interact with STAT1 [123]. The retention of STAT proteins in the cytoplasm is mediated by P1 and P2 proteins, which are excluded from the nucleus due to the NES signal. Indeed P1 alone can cause the cytoplasmic retention of STAT1, and treatment of cells with leptomycin B reverses or inhibits cytoplasmic sequestration of STAT1 [122]. The P3 isoform is also able to retain STAT1 in the cytoplasm using a distinct mechanism. P3 has been shown to interact with microtubules (MT) via a specific sequence MTAS, and this interaction mediates the sequestration of STAT proteins on the cytoplasmic MTs [125]. The capacity of P to inhibit nuclear translocation of STAT1 is conserved between the most distantly related members of the lyssavirus genus except for the attenuated strain derived from Nishigahara strain, NiCE for which P is mutated in the NES signal [126,127]. Consequently, P from NiCE is unable to be actively exported into the cytoplasm and subsequently inhibit STAT nuclear translocation and IFN signaling [127]. This illustrates the importance of active nuclear export of the RABV P protein in its ability to block IFN signaling and thereby in viral pathogenicity.

The P protein has also been reported to be able to inhibit the interaction of STAT1 with the target DNA, thereby inhibiting STAT1 signaling at an intranuclear stage [122]. This nuclear activity is presumably mediated by RABV P isoforms P3–P5 which are reported to localize into the nucleus [119]. Binding and sequestration of pSTAT1 in either subcellular compartment inhibits the interaction of pSTAT1 with ISRE promoter elements in the DNA, and subsequently impairs the pSTAT1-mediated expression of ISGs [122] (Figure 3). The ability of the P protein to target pSTAT1 in both of these compartments thus appears to be yet another demonstration of the functional efficiency of this protein. Accordingly, RABV infection before IFN treatment abolishes the capacity of this cytokine to induce the expression of ISGs [123]. Similar mechanisms of inhibition of STAT1 activity have also been described for the P proteins of the Hendra and Nipah Viruses (HeV and NiV, respectively) [128,129].

**Figure 4:**
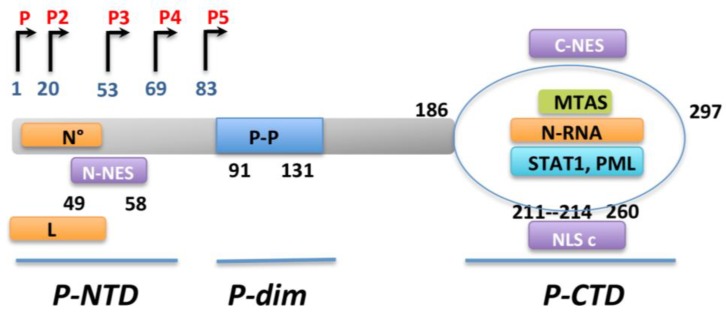
Functional and structural characterization of RABV-P protein. The RABV-P gene encodes a full length (P1) and four N-terminally truncated isoforms (P2-P5) from the initial and subsequent internal, in frame Met codons at indicated residues. RABV-P protein contains three functional domains separated by two intrinsically disordered regions: the N-terminal domain (*P-NTD*, residues 1 to 52), interacting with the soluble N protein (called N°) and L protein; the dimerization domain (*Pdim*, residues 91 to 131); and the C-terminal domain (*P-CTD*, residues 186 to 297), involved in binding to the N-RNA, as well as cellular proteins STAT1 and PML. The P protein also contains several targeting sequences including two NESs, a conformational NLSc, formed by the globular fold of the CTD.

### 5.5. Counteraction of PML Nuclear Bodies by RABV

During early viral infection, different viral proteins transiently colocalize with PML on NBs before disrupting them (reviewed in [75]). The functional consequences of the altered localization of the PML NBs could be a specific viral strategy to block cellular systems that may hamper viral replication.

PML NBs are not altered during VSV infection (unpublished data), whereas RABV infection reorganizes PML NBs, causing them to become larger and appear as dense aggregates [49]. Expression of RABV P (P1) can cause relocalization of one particular PML isoform, PMLIII from the NBs into cytoplasmic dots where both proteins colocalize. Interestingly, the expression of the P3 isoform, which lacks the dominant N-NES and can localize into the nucleus, results in reorganization of PML NBs analogous to that observed in RABV infected cells, indicating an intranuclear function of this P isoform and highlighting once again the importance of nuclear trafficking of the P products.

Both P1 and P3 bind to the RING finger motif of PMLIII in transfected or in infected cells, via a PML binding site in the CTD of P [49]. Thus, disrupting of PMLIII’s localization within the NBs could counteract its antiviral action. Accordingly, the anti-VSV and anti-RABV properties of PMLIV require its localization within the NBs [42,48].

## 6. Conclusions

In this manuscript, we have reviewed the mechanisms by which VSV and RABV trigger IFN production, the different ISG products that inhibit their lifecycle and the mechanisms by which these viruses counteract the IFN pathway. Elucidating the various strategies used by these viruses to usurp the cellular machinery for their own benefit will provide a better understanding of the relationship between the viruses and their hosts.

The functions of the majority of antiviral ISGs still remain unknown. In recent years, new antiviral ISGs have been identified and characterized for their contributions to intrinsic and innate immune antiviral activities. These ISGs encode distinct proteins with a diverse range of biological functions that directly block various stages of the viral life cycle. In addition, particular ISGs have also been implicated in innate immunity, by positively regulating IFN production, thus reinforcing viral resistance. Determining the mechanisms of antiviral ISGs and their implication in IFN response are main goals of future research. In cells treated with IFN, ISGs are the primary genes controlling the replication of viruses. Among the hundreds of known ISGs, only few have been validated *in vivo* for their implication in antiviral defense. While it is most likely that inhibition of the infection of any given virus by IFNs is through the induction of multiple ISGs that work cooperatively to disrupt a number of stages of viral replication, identification of individual antiviral ISGs and the subsequent elucidation of their modes of action are essential to uncover the antiviral mechanism of IFNs and viral pathogenesis. Understanding the intrinsic antiviral activity of the ISG products may introduce new ways for developing targeted antiviral therapy that would directly activate these genes and bypass the requirement for IFN treatment.
